# Optimized backpropagation neural network for risk prediction in corporate financial management

**DOI:** 10.1038/s41598-023-46528-8

**Published:** 2023-11-07

**Authors:** Lingzi Gu

**Affiliations:** School of Economics and Management, Wuhan Railway Vocational College of Technology, Wuhan, 430205 China

**Keywords:** Engineering, Mathematics and computing

## Abstract

Corporate financial management is responsible for constructing, optimizing, and modifying finance-related structures for an unremitting function. The finance optimization model incorporates risk prediction and fund balancing for distinguishable corporate operations. This risk prediction is handled using sophisticated computing models with artificial intelligence and machine learning for self-training and external learning. Therefore, this article introduces a Backpropagation-aided Neural Network for designing an Optimal Risk Prediction (ORP-BNN) to pre-validate existing and new financial imbalances. The risk prediction model is designed to cope with corporate standards and minimum riskless financial management. This is designed as a linear snowfall model wherein the BNN decides the significance between fund allocation and restraining. The snowfall model significantly relies on allocation or restraining, which is achieved by assigning significant weights depending on the previous financial decision outcome. The weight factor is determined using gradient loss functions associated with the computing model. The training process is pursued using different structural modifications used for successful financial management in the past. In particular, the risk thwarted financial planning using a snowfall-like computing model, and its data inputs are used for training optimization. Therefore, the proposed model's successful risk mitigation stands high under prompt decisions.

## Introduction

Every deal, fund, source, and capital income is managed in corporate financial management. Corporate finance maintains the activities, transactions, and interactions that enhance an organization's capital range^1^. Corporate finance creates, develops, and improves the financial structure of an organization or enterprise. Risk prediction is an important task to perform in corporate financial management systems. Risk prediction uses certain models and methods to predict challenges and problems during the financial management process^2^. Certain risk prediction methods are used in financial management systems. The backpropagation (BP) neural network is mostly used for risk prediction. A particle swarm optimization (PSO) algorithm is implemented in BP that predicts the exact risks presented in a corporation^3^. PSO increases risk prediction accuracy, enhancing the efficiency range of financial management systems. The embedded deep learning (DL) technique is also used in risk prediction methods^4^. The feature extraction method in DL extracts important patterns and features from the database. The extracted data produce optimal data for further prediction and detection processes. DL technique improves corporate financial management systems' performance and feasibility levels^5^.

Financial risk prediction is made in every organization and enterprise. Financial risk prediction is mainly used to predict management systems' risk factors and challenges^6^. The main aim of financial risk prediction is to increase accuracy and efficiency in decision-making. Computing models analyze the data required for prediction and detection^7^. The computing model studies the characteristics and features of the content presented in the database. Cloud computing (CC) based risk prediction methods are used in financial management systems^8^. CC identifies both autonomous and dynamic characteristics of risk factors, which are presented in the database. CC maximizes the overall accuracy of risk prediction, which enhances the efficiency and effectiveness level of financial systems^9^. The computing model provides necessary information for decision-making and architectural process that reduces energy consumption range in the computation process. Real-time risk factors and features are mostly predicted by a computing model that reduces latency in the decision-making process^10^.

Neural network (NN) algorithms and techniques are widely used for detection and prediction. NN algorithm predicts the exact content required to perform a certain task in an application or system. NN is also used in corporate financial management systems^11^. The NN-based prediction method is mostly used in financial management systems. A convolutional neural network (CNN) algorithm is used here to increase management systems' efficiency and reliability^12^. The feature extraction method is used in CNN, which extracts important features from the database. Feature extraction methods produce feasible data for detection and decision-making processes^13^. CNN method maximizes the accuracy in detection and prediction that improves the performance and significance levels of the systems. The back propagation neural network (BPNN) algorithm is also used in corporate financial management systems^14^. The BPNN algorithm detects capital problems, financial risks, and challenges. Hidden features and patterns of finance are also predicted by BPNN that reduce the error ratio in the decision-making process. The BPNN algorithm increases accuracy, providing better performance of management systems^15^. The existing systems are able to analyze the financial imbalance and risk problem effectively. However, the study difficult to address the fund balancing problems in the distinguishable corporate operations. The risk problem is addressed with the help of the Backpropagation-aided Neural Network for designing an Optimal Risk Prediction (ORP-BNN) to pre-validate existing and new financial imbalances. The contributions of the article are listed below:

- Designing an optimal risk prediction model for identifying financial imbalance of organizations for providing a better investment/ risk planning
- Employing back propagation learning model for linear snowfall analysis between distinguishable plan modifications for improving the decision precisions
- Performing a comparative analysis and data based analysis for validating the proposed model’s efficacy through comparison and data validations

## Related works

Kim et al.^16^ designed a data mining framework for financial prediction systems. This framework predicts the actual financial status and standard of markets. Real-time financial problems and challenges are also detected by data mining which reduces latency. The feature selection method used selects necessary patterns for prediction. The proposed framework maximizes effectiveness by increasing accuracy in the prediction process. Bias can be introduced during the selection of variables that have a major impact on the model. Different from this prediction method, Alhnaity et al.^17^ proposed a new hybrid intelligent forecast model for financial prediction. This is a hybrid model with three-step prediction that reduces the energy and time consumption ratio in financial forecasting systems. The prediction accuracy is optimized using genetic algorithm employed in this model. Data-driven approaches are useful, but they could miss unexpected or black swan events in the financial markets, which limits the model's capacity to foresee extreme events.

Colak et al.^18^ introduced a multivariate approach using balance sheets for assessing corporate financial risk. Multiple discriminant analysis is used here that analyze the variable which is required for prediction. Variables and characteristics are analyzed which provide feasible data for financial risk prediction. Financial strength and growth are improved by a multivariate approach. The introduced approach improves accuracy in detection and prediction processes for financial development systems. The study discusses the ability to predict, but it's crucial to take into account the models' interpretability because complicated indices may be difficult for stakeholders to comprehend and use successfully.

Yan et al.^19^ developed a deep learning (DL) framework for financial time series prediction. Long short-term memory (LSTM) algorithm and complementary ensemble empirical mode (CEEMD) are used in the DL framework that predicts the financial time series. The decomposition module exact necessary features from raw data that improve the prediction ratio. When compared with other methods, the proposed framework maximizes the robustness and efficiency range of financial systems. If the model doesn't take non-stationarity or abrupt changes in market conditions into account, its performance may be constrained.

Feng et al.^20^ designed a backpropagation (BP) neural network algorithm-based financial risk prediction model. The feature extraction method is used here that extract the important features from the database. The extracted features provide the necessary data for prediction and detection processes. The study focused only on limited time period for predicting financial crises.

Financial crises and issues are also predicted by the BP algorithm. Different from this concept, Zhao et al.^21^ proposed a corporate financial risk prediction-based embedded system. The main aim of the proposed system is to predict the financial cries and risks which are presented in corporations and enterprises. Embedded system identifies the operational and functional risks which cause various problems in enterprises. Embedded system maximizes the accuracy of risk prediction which enhances the efficiency of corporations. The proposed system increases performance and reduces the computation cost and time in enterprises.

Du et al.^22^ introduced a clustering-based under-sampling (CUS) heterogenous ensemble-based financial distress prediction model for imbalanced datasets. An ensemble feature selection technique is used here that selects the necessary data which are required for prediction. Gradient boosting decision tree (GBDT) is also used in prediction models that classify the types of financial distress. The datasets utilized in this research might include certain limitations due to the unique ownership arrangement and management framework of publicly traded businesses in China.

Another version of GBDT is introduced by Qian et al.^23^. The authors developed a new financial distress prediction method based on GBDT. An ensemble learning algorithm is used in the prediction method that maximizes the accuracy of the prediction process. Feature selection technique is also used in the proposed method that extracts the important features from given datasets. Feature selection reduces both the time and energy consumption ratio in computation which improves the efficiency of the systems. The proposed method increases accuracy in financial distress prediction which enhances the performance of corporations. Performance of the model could be constrained by biased or insufficient data.

Cui et al.^24^ developed a web-embedded system (WES) based internet financial risk prediction model. The main aim of the proposed model is to reduce the error ratio in the decision-making process. An analysis system is used here that analyzes important features and behaviors of financial risks which provides optimal data for further prediction and detection processes. A hybrid model is introduced by Paquet et al.^25^ for the financial risk prediction process. An encoder is embedded in a neural network that transfers time series into density matrices which provide relevant data for financial prediction. Quantum neural is mainly used here to trace the risk based on certain functions and conditions. When compared with other traditional methods, the proposed method maximizes the accuracy and effectiveness range in financial prediction. The extrapolation regime is limited to 20 days in the study and investment methods sometimes entail longer time horizons, it is crucial to examine the system's performance throughout these intervals.

Clintworth et al.^26^ introduced a financial risk assessment for shipping. The main aim of the proposed method is to identify financial risks and distress which are presented in industries. A holistic machine learning algorithm is used here that predicts actual data which are necessary for evaluation and prediction processes. The proposed method maximizes the accuracy of the distress prediction process. Experimental results show that the proposed method increases the development and efficiency levels of industries. It's critical to evaluate the methodology's stability and robustness over time and in the face of shifting market conditions.

Huang et al.^27^ designed a financial distress prediction method based on textual sentiment. Audit reports and analyses are conducted that provide textual sentiment data for the prediction process. The extracted sentiment data produce annual reports of an organization. Both credit risks and issues are detected by the prediction that reduces the error ratio in the decision-making process. Lack of considerations for credit risk assessment and handling risks.

However, a novel improvement is required for improving the prediction accuracy as defined in the following work. Xu et al.^28^ developed a deep learning (DL) based financial time series prediction method. A convolutional neural network (CNN) algorithm is used here that increases the overall accuracy of the prediction process. Feature extraction is implemented in CNN which extracts important features and patterns from the original for prediction. Feature extraction reduces both time and energy consumption range in the prediction process. Experimental results show that the proposed DL method enhances the efficiency, robustness, and reliability of financial applications. Lack of focus for outside factors like economic developments or changes in policy.

Li et al.^29^ introduced a backpropagation neural network (BPNN) based financial risk prediction. The proposed BPNN method achieves high accuracy in financial risk prediction. BP detects the risks at an early stage which reduces unwanted problems in industries and enterprises. Operational principles and patterns are extracted from databases that provide necessary data for the risk prediction process. Lack of Decision-making benefit from knowing the causes of estimates of bankruptcy risk.

The training issues in this method are addressed by Wu et al.’s^30^ proposal for excessive financialization risk evaluation method using machine learning (ML) for trading enterprises. A support vector machine (SVM) algorithm is implemented in the evaluation method that evaluates the accuracy ratio in risk prediction. SVM trains the datasets which are required for the evaluation process. Experimental results show that the proposed evaluation method enhances the accuracy of decision-making which improves the efficiency of trading enterprises. Internet finance prediction can vary from others and generalizability was not addressed.

Yao et al.^31^ proposed a new relationship analysis model for enterprises. The main aim of the model is to analyze the relation among social responsibilities, debt financing cost, and enterprise innovation level. It creates a major impact to improve the performance range by comparing the relationships among the factors. The proposed model helps enterprises to increase the degree of competition at market levels. The result of immediate application of Hexun, which is a reasonably simple is a drawback in corporate social responsibility measurement tool.

Panjaitan et al.^32^ developed an evaluation method to understand the decisions that are made by management systems in heavy industries. The developed method analyzes the determinants of the decision-making process which reduces the complexity of the management process. It provides limited access to cash capital that enhances the effectiveness level of the emission reduction process. The developed method reduces the computational cost range of heavy industries. Additionally, in such economies, the role of stakeholder groups is nevertheless not intended to support corporate actors' commitment to climate change.

Lahouel et al.^33^ introduced a regime-switching model for enterprises. The introduced model evaluates the relationship between financial performance and the social responsibilities of the industries. The introduced model uses an inverted U-shaped network to analyze the feasibility range of the industries. It eliminates the nonlinear negative features from the relationships. The introduced model improves the overall theoretical relationship ratio among the determinants. Interpreting models that are not linear can be difficult.

Based on the above related works financial world had been transformed with their unique research ideas and paved the way for a safer, more predictable financial landscape. The discussion addresses issues in financial management and optimization, underscoring the significance of risk prediction and fund allocation in corporate operations. Together the existing works unveiled the hidden treasures of accuracy, reduced risks, and enhanced the efficiency of the financial world. Even though, the existing work has lots of advantages, still room for an improvement like resolving lack of transparency, challenging decision making, issues in creating comprehensive risk management system, poor generalization to new data, and financial risk prediction. These limitations highlight the need for a balanced approach that combines advanced techniques with real-world data to create effective financial risk management solutions. Hence three algorithms from existing work have been chosen including CUS + GBDT^22^, DMT-FP^16^, QuantumLeap^25^ for comparison purpose to show the efficacy of proposed ORP-BNN approach.

## Methods

### Optimal risk prediction using backpropagation neural networks

Uncertainty in earnings and cash flows in any society faced some risk factors based on the risk prediction can be computed. This risk factor varies from financial risks and types of product price risks in different aspects. First, risk prediction is an investment risk that impacts shares and operations, not the product price. Second, fund allocation is highly risky because every commodity's investment and shares can vary. Third, local risk weight is less related to the financial management risk relying on investment and share rate risks. Due to increasing fund allocation and restraining, the organizations started to aid risk prediction to measure the adverse impacts of demand fluctuations caused by uncertain investments, shares, and operations. This problem is addressed using the snowfall decision model to identify the imbalance of fund allocation and risk weight. Corporate financial management generally answers snowfall risks by modifying investments, shares, and operations that are directly found out the risk, like slope down or snowfall making, to alleviate the modifications in snowfall. Based on the enormous population growth finance optimization model incorporates corporate standards, geographical diversification, and fund allocation and has proceeded as a better approach for minimum riskless financial management. On the company level, the effect of poor snow conditions on one organization can be alleviated by disseminating the risk to other organizations within the society and channeling capital and resources with other organizations. In common, operational hedges such as snowmaking (fund allocation) and geographical diversification (financial imbalance) obtains for capital investment and shares and cannot be retrieved easily. The financial imbalance occurs if the investment streams fail to meet the returns/ income regardless of the shares planning or any other investment. A prolonged imbalance results in organizational loss incurring market value drops.

### Analyzing the effects of financial imbalance on risk prediction

**Figure 1:**
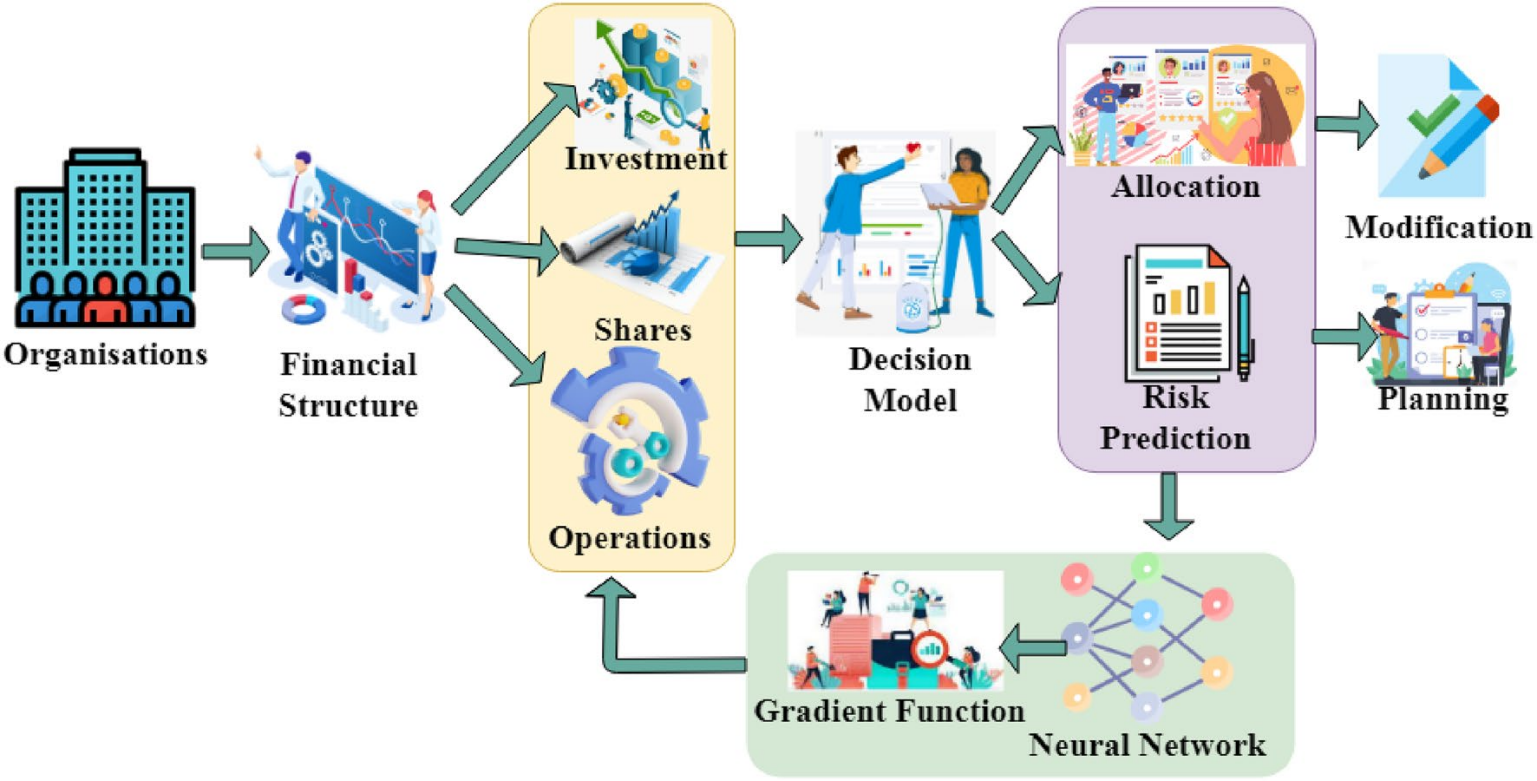
Proposed ORP-BNN model.

This section measures the adverse impacts of financial imbalance using the snowfall decision model for risk prediction in two scenarios: (1) single organization versus multiple organizations and (2) existing versus post-adding new financial imbalances to the different organizations' financial structure. Previous studies on the impacts of financial imbalance risk exhibited the outputs in inconsistent solutions. Examine operational hedges' impact on corporate financial management's investment and share rate forecasts. The existing fund allocation and financial imbalance in a particular organization is less chance to exhibit investment risk. Contrarily, high financial imbalances between all the organizations have relatively high gradient loss due to assigning weights for allocation and restraining. An inconsistency in fund allocation contains investment risk from the modification rate and share risk from the uncertain demand in foreign units. At the same time, fund-related modification risk can be mitigated by financial imbalance and share risk until it remains due to variations in local demand. Figure 1 presents the proposed model.

In the context of this study, snowfall risk affects only the investment, not the price of shares, cash flows, and earnings. The BNN is responsible for measuring the importance of fund allocations and restraining that improves corporate financial management for enhancing the organizations through risk prediction. The risk prediction is performed based on demand changes and financial imbalance in organizations, whereas the investment, shares, and operations are the financial decision outcome made by the weight factor. The number of organizations ${Org}_{n}$ and their financial structure ${F}_{s}$ are the serving inputs for improving corporate financial management. The finance optimization and risk prediction for single or multiple organizations are measured for distinguishable corporate operations. The financial structure is classified into three types: investment, shares, and operations. The corporate management function differs for all organizations relying on employees and manufacturing products. The organization is to handle the financial data and many users using sophisticated computing models. First, risk prediction is keen in corporate financial management for an unremitting function is expressed as

1 $$\left.\begin{array}{c}\underset{N\in i}{\mathrm{max}}{Org}_{n} \forall {F}_{s}\\ Where\\ {F}_{s}=\left(Inv+Shr+Opt\right)\\ \underset{j\in \mathit{Dm}}{\mathrm{min}}{R}_{P}\forall \left(Inv+Shr\right)\end{array}\right\}$$

Such that,

2 $$\left.\begin{array}{c}{R}_{P}={Org}_{n}\left[{Inv}_{r}-{Shr}_{r}\right]\\ and\\ \underset{N\in i}{\mathrm{min}}{F}_{{s}_{i}} \forall N\in {C}_{Opt}\end{array}\right\}$$

As per Eqs. (1) and (2), the variables $Inv$, $Shr,$ and $Opt$ used to represent corporate financial management working for $N$ tasks based on investment, shares, and operations. In the next organization's financial structure representation, the variables ${R}_{P}$, ${Inv}_{r}$ and ${Shr}_{r}$ Illustrates risk prediction, investment rate, and share rate, respectively. The second objective is to manage the minimum riskless financial management using risk occurrence prediction is validated using the condition ${Org}_{n} \forall N\in {R}_{P}$. If $Emp=\left\{\mathrm{1,2},\dots , emp\right\}$ represents a set of employees in the organizations for pre-validation of existing and new financial imbalance, then the number of fund allocation and restraining in the financial management at processing time interval $t$ is ${F}_{alloc}\times t$. The fund allocation based on demands and corporate operations with $emp\times {F}_{alloc}$ and ${F}_{alloc}\times r$ is the available financial structure. Risk prediction planning and fund allocation modifications are optimized and computed using a linear snowfall model for analyzing the financial imbalance. In this analysis, the decision model is classified to verify the modifications in BNN. The country's financial management depends on people's demands and requirements for sustaining the corporation's standards. Based on the corporate standard $\left({C}_{s}\right)$, the $N$ users, organizers, stakeholders, and partners' availability in a single organization is fetched. The additional processing time is needed for modifying the fund allocation and minimum riskless financial management to improve user needs and requirements. The existing and new financial imbalances are validated for further fund allocation modifications and identified using a machine learning process for external learning and self-training. After the fund imbalance validation is performed, risk prediction is the significant feature for assigning weights. From this weight factor computation, the snowfall model relies on either allocation or restraining is achieved by assigning significant weight based on the previous financial decision outcome. The prevailing instance of determining risk prediction in single or multiple organizations is analyzed. The modifications in fund allocation are achieved through BNN for considering the user needs are requirements in the following section. The financial structure considered is induced for a decision model for identifying the optimal risks and the investment allocations accordingly. Both prediction and allocation processes are identified from the decisions based on previous and current changes in the actual structure.

The functions of the risk prediction model are augmented in this section. The prediction model is designed for allocation and detecting financial imbalance across various investment intervals. In this risk prediction model, the continuous identification of investment rate risk and share rate risk is validated for every organization with the ORP-BNN model and ${F}_{alloc}\times t$ is analyzed for validating the fund allocation for all ${Org}_{n}$ and ${F}_{Imb}$ is the considering factor. The probability of risk factor prediction ${\rho }_{{R}_{P}}$ and financial imbalance ${\rho }_{{F}_{Imb}}$ in corporate financial management is expressed as

3 $${\rho }_{{R}_{P}}={\left( 1-{\rho }_{{Org}_{n}}\right)}^{t-1} , N\in t$$

where,

4 $${\rho }_{{Org}_{n}}=\left(\frac{{F}_{alloc}\in N}{{F}_{alloc}\in t}-{R}_{P}\right)$$

Instead,

5 $${\rho }_{{F}_{Imb}}=\left(1-\frac{{R}_{P}\in N}{{R}_{P}\in t}\right)*{Org}_{n}$$

**Figure 2:**
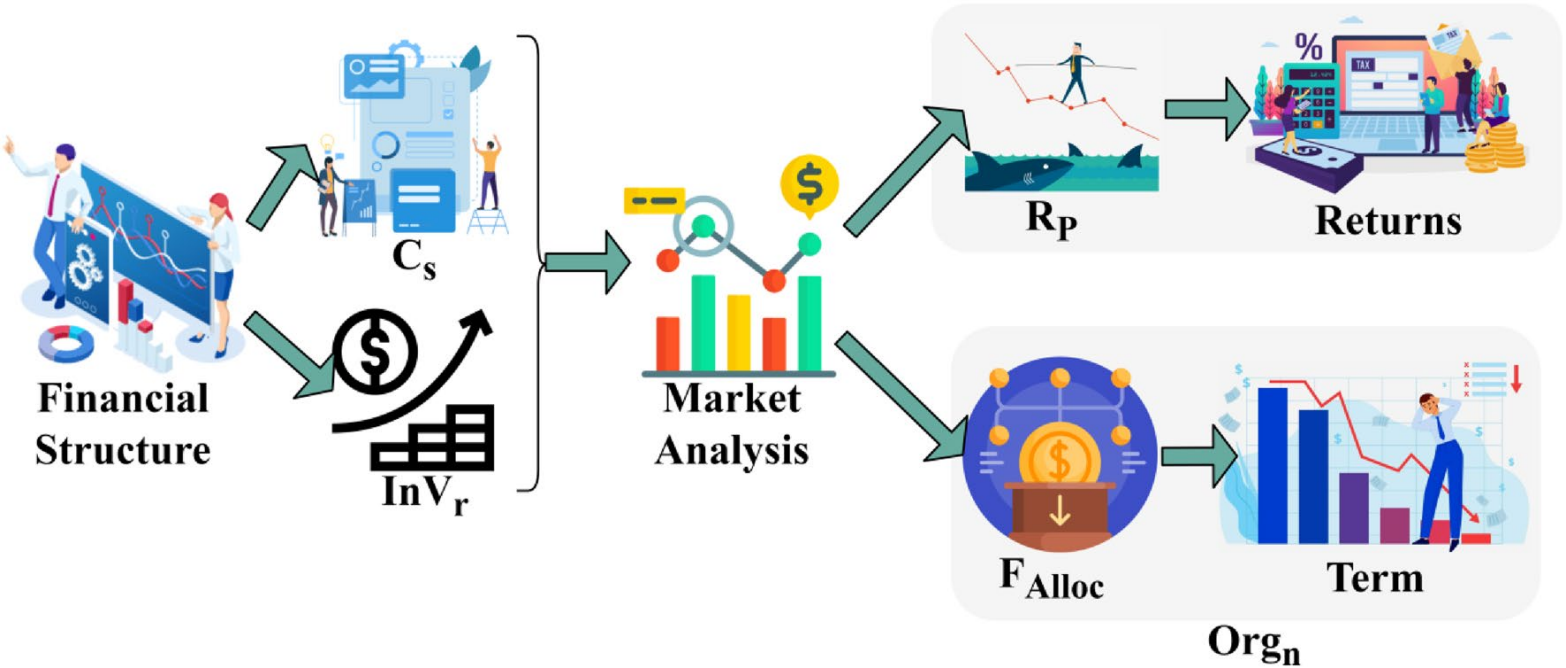
Risk factor and imbalance prediction process.

As per the above Eqs. (3), (4), and (5), the continuous fund allocation from the organizations and those facing financial imbalance is measured based on the corporate standard. Therefore, there are no pending user needs and requirements. The risk factor and imbalance prediction process are illustrated in Fig. 2.

The financial structure is divided into ${C}_{s}$ and $In{V}_{r} \forall T$ and $N$ such that the market analysis is performed across different factors. Initially the $Or{g}_{n}$ and $Opt$ are the dividends for ${R}_{P}$ and ${F}_{imp}$. The ${R}_{P}$ is an external concern whereas ${F}_{imb}$ relies on internal $Opt$. Therefore the terms $T$ and returns are mandatory for identifying risks and financial imbalance (refer to Fig. 2). Hence, the financial structure assessment is substituted as in Eq. (1). Therefore, risk occurrence prediction in ${\rho }_{{Org}_{n}}$ follows

6 $$Risk\, Prediction\,\left(N\right)=\frac{1}{\left|{Inv}_{r}+{Shr}_{r}-1\right|} .{\left({\rho }_{{Org}_{n}}\right)}_{t} ,\quad  N\in t$$

In Eq. (6), the significant snowfall model for $N$ users as per Eq. (6) is achieved in fund allocation and restraining to ensure precise financial decision outcomes. The significance between fund allocation and restraining is analyzed for assigning accurate weight using BNN at different $t$ intervals to reduce user demand fluctuation's impact on the organization. For the condition $\left(N\times {F}_{alloc}\right)>\left({F}_{alloc}\times t\right)$, the risk prediction is descriptive using BNN and organization convenience. If a high-risk occurrence is predicted in a single organization, the weight factor is assigned to alert that organization and others. Hence, the weight factor is determined using gradient loss functions associated with the evaluation model and follows $N>t$ and ${\rho }_{{Org}_{n}}$ leads to minimum riskless financial management and satisfies the above Eq. (1). Several financial decision outcomes rely on prolonging ${\rho }_{{Org}_{n}}$ for reliable fund allocation. Hence, the fund allocation and restraining outputs are based on geographical diversification with risk prediction for recurrent modifications. The modifications are performed depending on the different corporate structure requirements. In the requirement assessing process the maximum investment and risk computing variants are validated.

In corporate financial management, the finance-related structure construction, optimization, and medication are validated using the condition $N\times {Inv}_{r}$ is achieving maximum investment and hence high risk, and the processing time is invariant. The high and low risks in corporate financial management are identified along with the corporate standard for fund allocation and restraining. The risk prediction planning and fund allocation modifications are the deciding factors here. The probability of individual organization risk $\left({\rho }_{{in}_{Rk}}\right)$ is computed as

7 $${\rho }_{{in}_{Rk}}=\frac{{\rho }_{{R}_{P}}. Risk\, Prediction\left(N\right).\left[\left({Inv}_{r}-{Shr}_{r}\right)*{\rho }_{{Org}_{n}}-\left(\frac{{F}_{alloc}-res}{N}\right)\right]f\left(GrL\right)}{f\left(GrL\right).t}$$

where,

8 $$f\left(GrL\right)=\sum_{N=1}\frac{\left({Inv}_{r}-{Shr}_{r}\right)*{F}_{alloc}*{\rho }_{{R}_{P}}}{Risk\, Prediction\left(N\right)}$$

**Figure 3:**
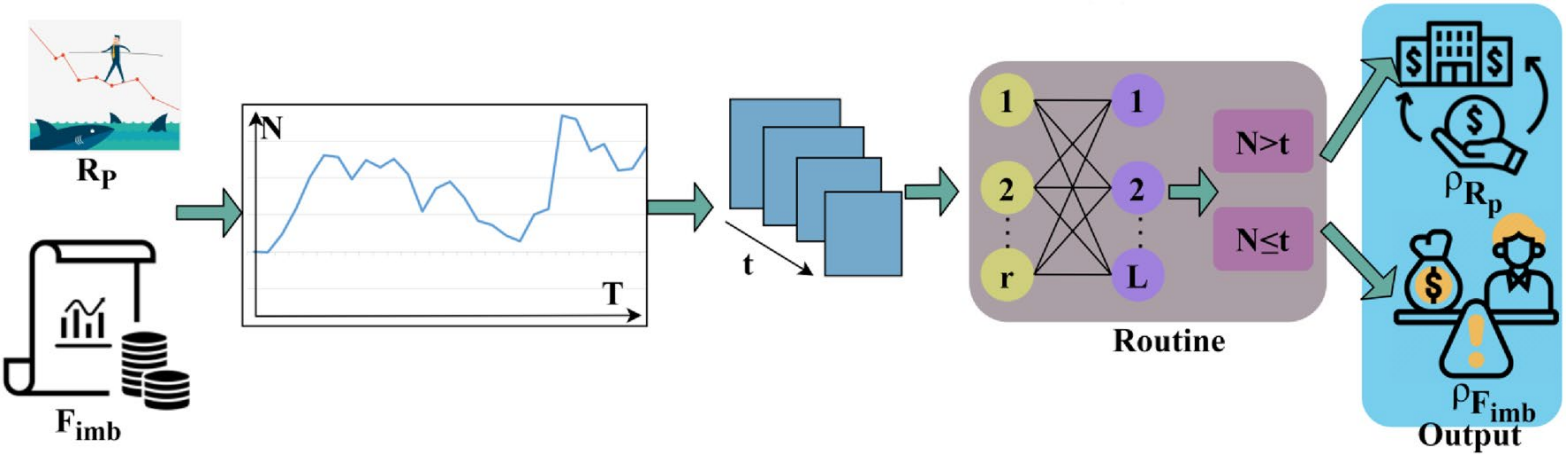
Routine analysis of $RiskPrediction (N)$.

As per Eqs. (7) and (8), the variable $f\left(GrL\right)$ denotes the gradient loss function performed at different $T$ intervals. From the risk prediction planning, the uncertain demands, needs, and requirements of the organization's users are identified for managing sustainability. The risk identification based on the above condition obtains a high weight factor and increases the demands and needs. The routine analysis for $RiskPrediction(N)$ is portrayed in Fig. 3.

Routine analysis is required to identify ${\rho }_{{R}_{p}}$ and ${\rho }_{{F}_{imp}}$ using the actual $\left(N\times T\right)$. This $\left(N\times T\right)\forall t$ is validated for $r$ and $L$ using all possibilities. The possibilities are classified for $N>t$ and $N\le t$ such that ${\rho }_{{R}_{P}}$ is extracted from $\left(In{V}_{r}-Sh{r}_{r}\right)$ and ${\rho }_{{F}_{imb}}$ is extracted from $({F}_{alloc}-res)$. Therefore the routine is repeated from $(r\times L)$ for identifying new $N$ across risk prediction (N) (Fig. 3). From this routine analysis of corporate financial management, the fund allocation modification and decision outcomes rely on identifying the high and low risks in organizations for processing and validating time are the deciding factors. These factors are computed using machine learning to mitigate the adverse impact of uncertain demands and needs. The following section describes the fund allocation modification and training process performed using multiple structural modifications for successful financial management. In particular, the risk of thwarted financial planning is processed through a snowfall-like computing model. The associated data inputs are used for training finance optimization. Pursued by the optimization, the modifications in finance allocations are required for improving the decisions across various risks encountered. This allocation modification is discussed in the below subsection.

#### Allocation modification

The financial decision outcome is used for classifying risk planning using BNN. It is processed for fund allocation and restraining the corporate financial management for single and multiple organizations. Using machine learning, the risk prediction planning is validated for further fund allocation modifications with certain user demands and requirements. The classification is performed for financial structures to identify the risks and gradient loss function probabilities at a similar time of risk prediction. Therefore, the condition for risk prediction is different for all organization that follows distinguishable corporate operations through financial planning. The training optimization is performed to mitigate the risk factor based on the available structures and rate analysis for further computation. The first risk prediction relies on high investment $\left({Inv}_{r}\right)$, $\left({Shr}_{r}\right)$ and $f\left(GrL\right)$ is expressed as

9 $$f\left(GrL,{Inv}_{r}\right)=\left[{Shr}_{r}-\left(\frac{{\rho }_{Org}}{{\rho }_{{R}_{P}}}\right) \times \frac{t}{N}\right]-Risk\, Prediction\left(N\right)+1$$

In Eq. (9), the financial planning depending on risk prediction in fund allocation for all organizations and its successful financial management is identified as per an analysis of individual ${\rho }_{Org}$ and $Risk Prediction\left(N\right)$. Here, the chances of the gradient loss function are used for less risk occurrence in the distinct organization achieving continuous fund allocation for improving financial management. Hence it is expressed as

10 $${\rho }_{Org}(t/{\rho }_{{R}_{P}})=\frac{1}{\sqrt{2N}}experssion\left[\frac{{Inv}_{r}-{\rho }_{{in}_{Rk}}\times Opt}{t}\right]$$

From the above Eq. (10) probability of training optimization computation through financial planning, the objective is to balance fund allocation and restraining. This balance function minimizes the processing time and risk factor. Hence, the actual fund allocation of a particular organization is computed as

11 $${F}_{alloc}=\mathrm{max}\left[\frac{{Inv}_{r}\times {Shr}_{r}\times Opt}{Risk\, Prediction\left(N\right)-GrL}\right]$$

**Figure 4:**
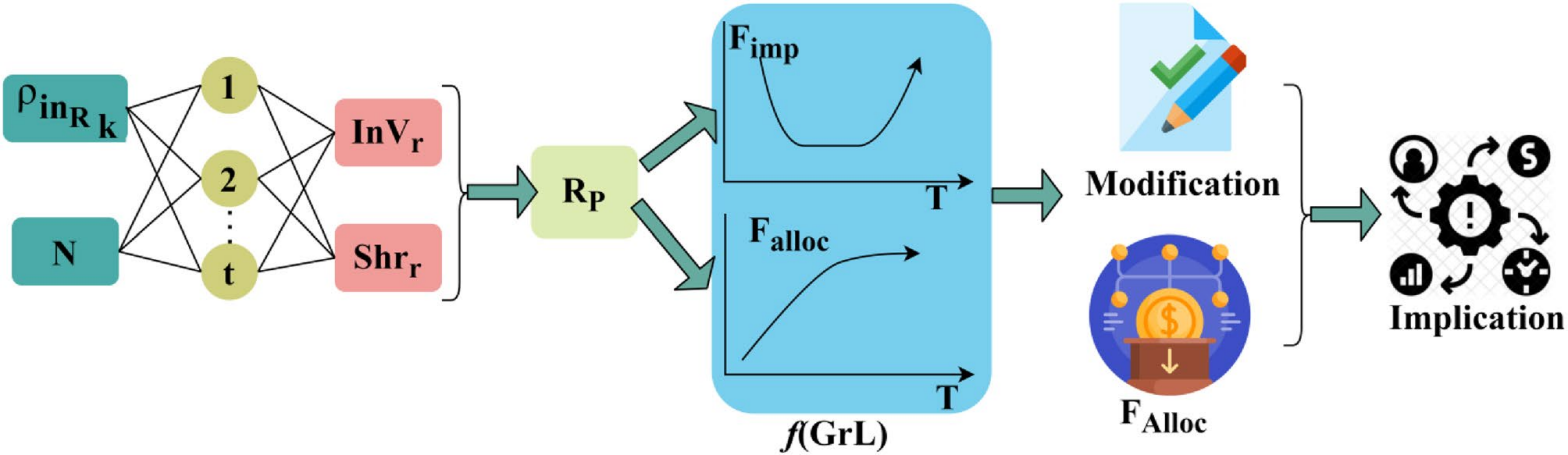
Allocation modification/planning illustration.

In Eq. (11), the fund allocation of an individual organization and financial decision outcome is computed. The risk prediction in corporate financial management through the snowfall computing model is to minimize the flaws and losses at the time of fund allocation. The allocation modification/ planning is illustrated in Fig. 4.

The ${\rho }_{i{n}_{{R}_{k}}}$ and $N\in T \forall t$ from the $BNN$ is used for providing modification (or) ${F}_{alloc}$. In this process the ${F}_{imb}$ and ${F}_{Alloc}\forall t$ (smaller split) $T$ is analyzed. This analysis is a gradient-less validation $\forall {\rho }_{org}(t/{\rho }_{{R}_{P}} )$ throughout the previous ${\rho }_{{R}_{p}}$. In the neural network analysis, only the downfall for ${F}_{alloc}$ is considered, and hence $Sh{r}_{r}$ and $In{V}_{r}$ are modified (Refer to Fig. 4).

The exceeding uncertain investment and shares outputs in high risk and then training optimization is provided for that organization then the processing time is demanding increases. Different structural modification is performed for successful financial management at different $T$ intervals based on the user needs and demands. Here, the processing time and risk prediction from an individual organization are the additional metrics fund allocation and restraining for both single and multiple organizations does not augment gradient loss and flaws. Therefore, corporate financial management is employed for managing the standard, and risk prediction is planned to analyze the input data without increasing the loss and reducing flaws.

## Results and discussion

**Figure 5:**
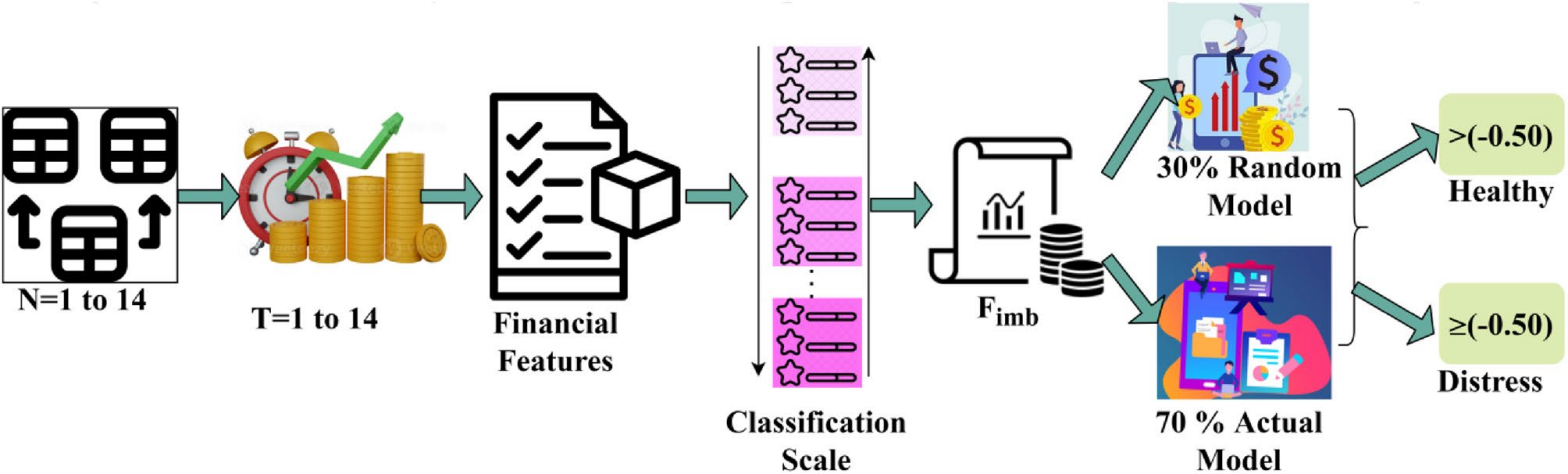
Financial classification based on input data.

The proposed model's analysis is performed using the^34^ dataset, which provides 80 fields and 3673 entries of finance data. The observation is performed between 1 and 14 sequences for 14 organizations. The analysis for financial risk prediction/ distress is represented in Fig. 5.

**Figure 6:**
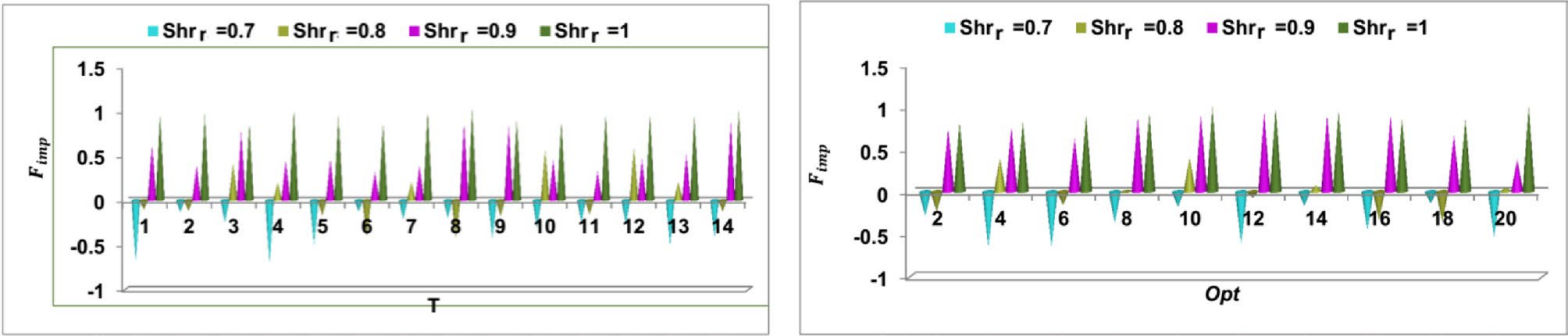
$${F}_{imb}$$ for $$T$$ and $$Opt$$.

The available data is used for classifying the scale across different financial features. This scale is used for ${F}_{imb}$ detection using 30% random and 70% actual models under varying inputs. The $>\left(-0.50\right){F}_{imb}$ the organization is considered as healthy, whereas the ones less than the above value are considered are under risk. Such risks are addressed using new models before the successive $T(>1)$ (Refer to Fig. 5). As mentioned in the dataset, the ${F}_{imb}$ is the deciding factor for $In{V}_{r}$ (or) $Sh{r}_{r}$. Therefore, for the varying $T$ and $Opt$, the ${F}_{imb}$ is analyzed as in Fig. 6.

**Figure 7:**
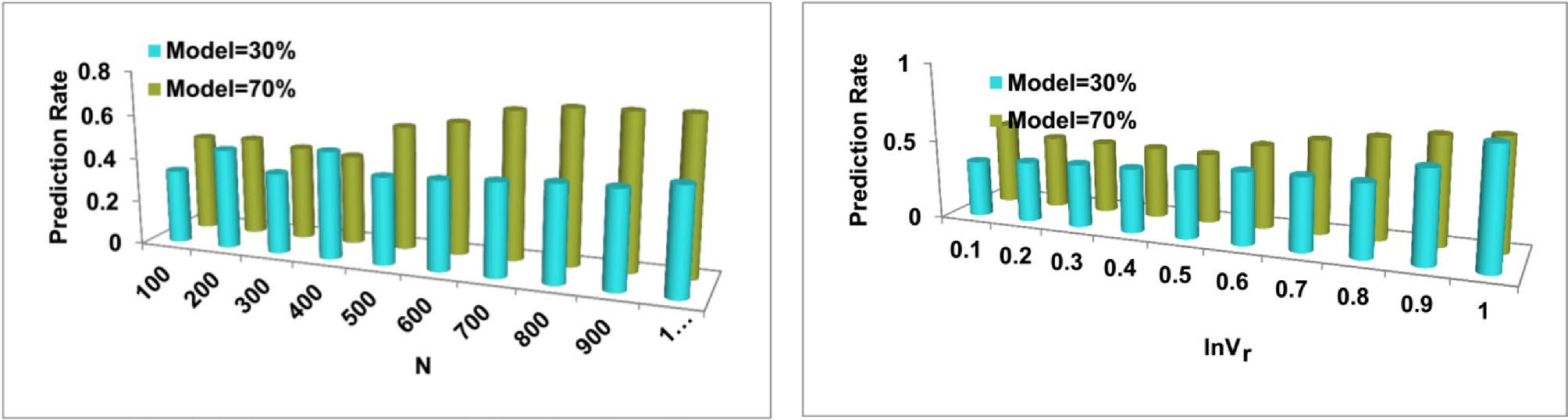
${R}_{p}$ for random and actual models.

The analysis is performed under varying $$f(GrL)$$ provided $${C}_{s}$$ is retained. In both $$T$$ and $$Opt$$ analysis, the impact of $$ {F}_{imb}$$ varies $$\forall Sh{r}_{r}$$. As the $${F}_{imb}$$ reduces the $$Sh{r}_{r}$$ increases or vice versa. Considering the $$RNN$$ arrangement, the $$f\left(GrL\right)$$ is modified for tuning the new parameter. This parameter is required for $${{\rho }_{Org}}_{n}$$ is Risk Prediction $$(N)$$ such that $${F}_{imb}$$ is confined. Post the suppression of $${F}_{imb}$$ further investments are drawn with the chances of Opt expansion or $${\rho }_{i{n}_{{R}_{k}}}$$. This is therefore analyzed for identifying a new investment policy for upgrading the $${F}_{imb}$$ in the consecutive $$T$$ (Fig. 6). Now, the $${R}_{P}$$ under the random and actual model $$\left({F}_{Alloc}\right)$$ is analyzed from the $${F}_{imb}$$ outputs as in Fig. 7.

**Figure 8:**
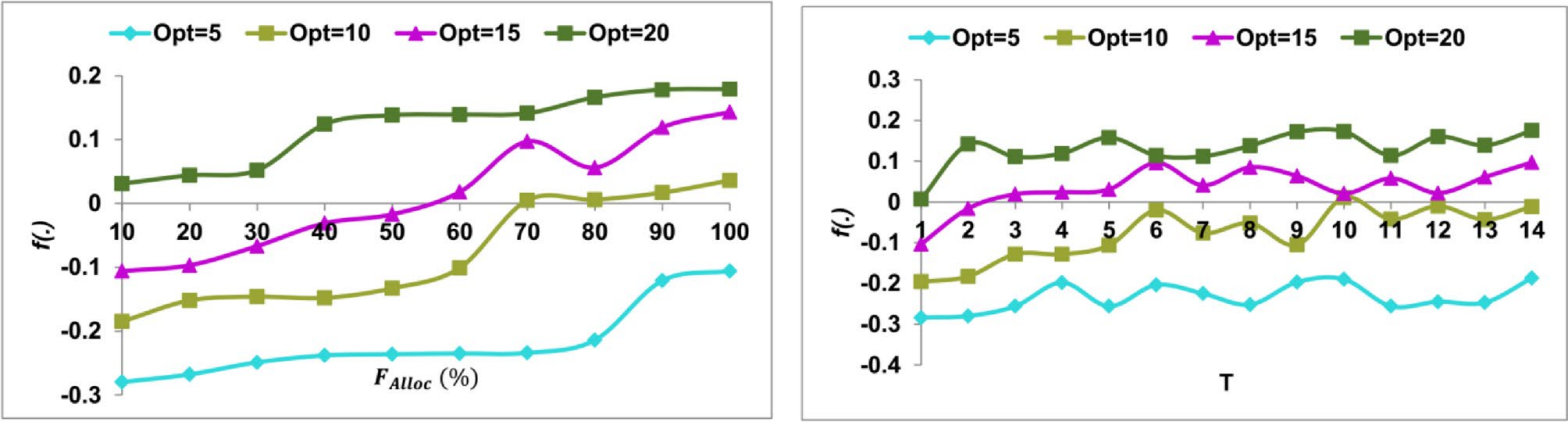
$$f(.)$$ for $$N$$ and $${f}_{Alloc}$$.

The $${R}_{p}$$ under $$N$$ and $$In{V}_{r}$$ have a different impact from the actual and random $${F}_{Alloc}$$ models. Depending on the available inputs and the previous $${F}_{imb}$$ the rate is identified. Considering the $$f(GrL)$$, the availability and deviations are suppressed. This process relies on the decision models exclusive for $$In{V}_{r}, Sh{r}_{r},$$ and $$Opt$$. As the expansion increases the $${R}_{p}$$ for multiple $$ {P}_{Or{g}_{n}}$$ increases and hence $${R}_{P}$$ influence is high. This BNN deviates the simplification through $$f\left(GrL, In{V}_{r}\right)$$ for now $${F}_{Alloc}$$. Therefore the analysis for $$f\left(GrL, In{V}_{r}\right)$$ is required for which before and after suppression is required. This is presented in Fig. 8.

The $f\left(.\right)$ relies on $N$ and ${f}_{Alloc}$ such that the first risk prediction is analyzed for new ${f}_{Alloc}$. Considering the $Sh{r}_{r}$ and ${C}_{s}$ for the varying ${\rho }_{{R}_{p}}$ increases the $f\left(.\right)$ across multiple ${\rho }_{or{g}_{n}}$. This is utilized for $T$ consecutive sequence such that ${\rho }_{org}(t/{\rho }_{{R}_{P}})$ is utilized. In this utilization the ${F}_{Alloc}$ is required to prevent ${F}_{imb}$. Therefore the $f(.)$ is required for preventing multiple Risk Prediction $(N),$ and hence $f(.)$ is suppressed at the initial trial. This enhances the optimal risk prediction across distinguishable $N$ (Fig. 8). Apart from the above discussion, a comparative study is presented in the following section. The metrics prediction rate, imbalance detection, modification recommendation, computing time, and model overhead are analyzed for the varying allocations and modification rate. The alongside comparison methods are CUS + GBDT^22^, DMF-FP^16^, and QuantumLeap^25^.

### Prediction rate

**Figure 9:**
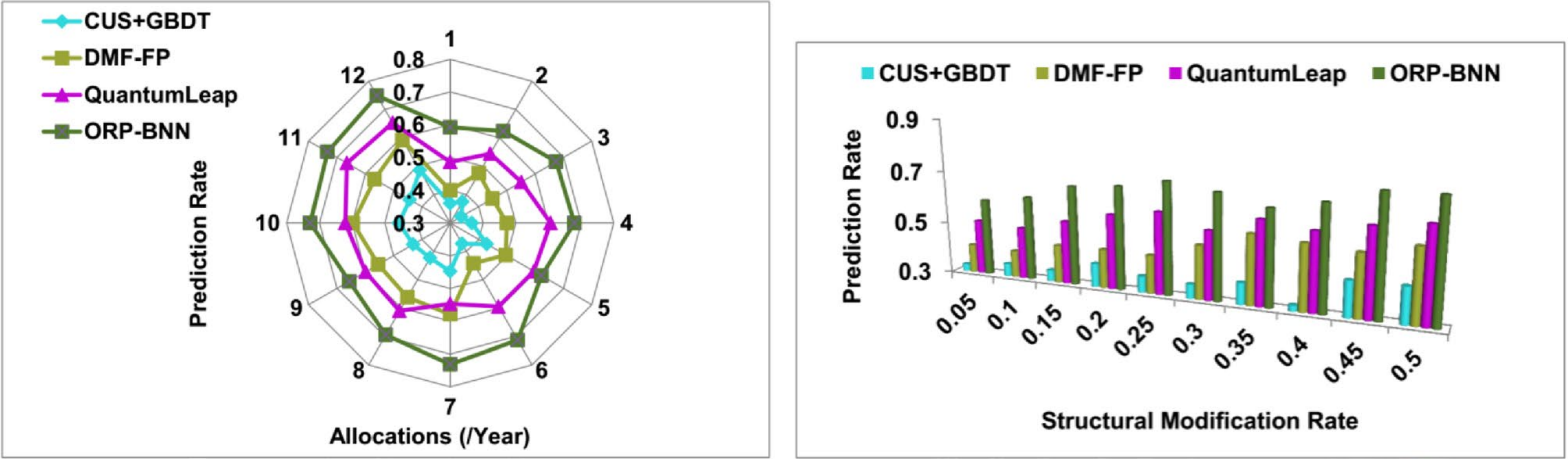
Prediction rate analysis.

In this, corporate financial management satisfies high-risk prediction rate for deciding the fund allocation and restraining using a snowfall-like computing model (Refer to Fig. 9). The analysis of input data and finance-related structure is balanced based on the above conditions for better modification recommendations due to risk occurred financial management. The existing and new financial imbalances are measured through users' demand and interest in that organization. From the existing organization, financial structure analysis is used for identifying the model overhead for both conditions. Instead, the computing time is evaluated for maximizing the prediction rate for risk occurrence along with input data; hence modification recommendation is increased. In the single or multiple organizational-based data analysis, the users' demands and requirements are identified for preventing model overhead in corporate financial management. Therefore, the investment rate and share rate is to be vary based on $\left(\frac{{F}_{alloc}\in N}{{F}_{alloc}\in t}-{R}_{P}\right)$, then the modification recommendation is provided for retaining the imbalance factor and financial decision outcome. In the proposed model, the data inputs are analyzed for training finance optimization then a new financial structure is generated to minimize the risk occurrence.

### Imbalance detection

**Figure 10:**
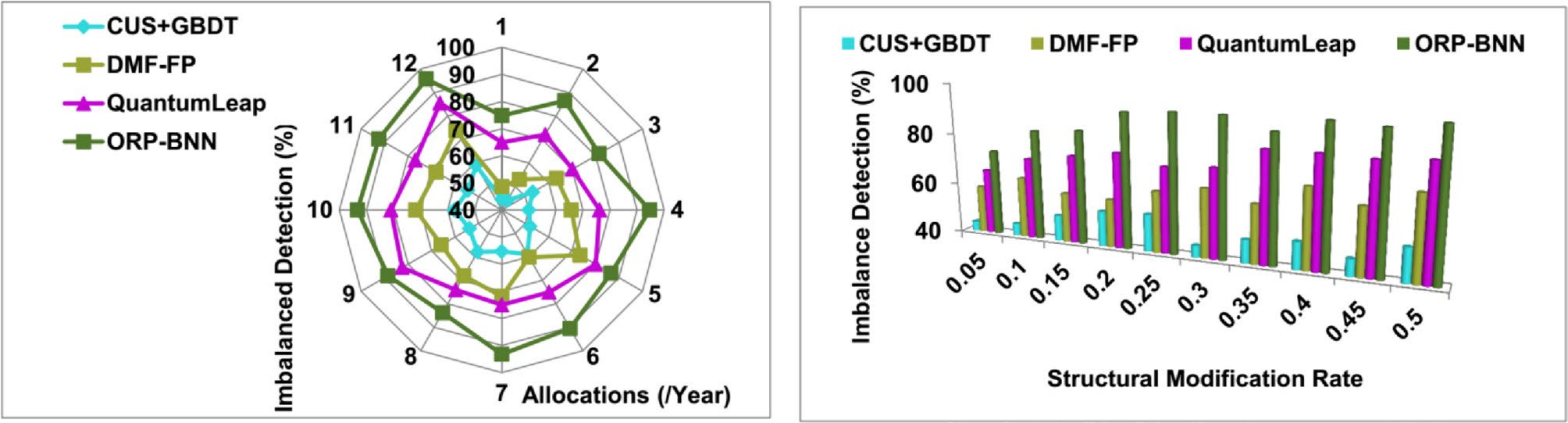
Imbalance detection analysis.

The data input analysis is high in this proposed model to identify constructing, modifying, and optimizing financial structures compared to the other factors in corporate financial management based on user needs and demands (Refer to Fig. 10). In this manuscript, the artificial intelligence and machine learning are used for identifying financial imbalance; hence self-training and external learning is achieved at different time intervals. The above two conditions are based on user demands and recommendations along with financial structure for increasing corporate standards [as in Eqs. (7) and (8)]. The fund allocation and risk prediction planning are continuously analyzed for accurate modification recommendations to be achieved. Based on the BNN and linear snowfall model, input data analysis is performed for different structural modifications. The maximum financial imbalance identified in this finance optimization model outputs in high-risk weight, investment rate risk, and share rate risk; it is considered for better fund balancing and risk prediction. In this proposed model, the training optimization depends on financial structures, and therefore, different structural modifications are performed to reduce risk occurrence.

### Modification recommendation

**Figure 11:**
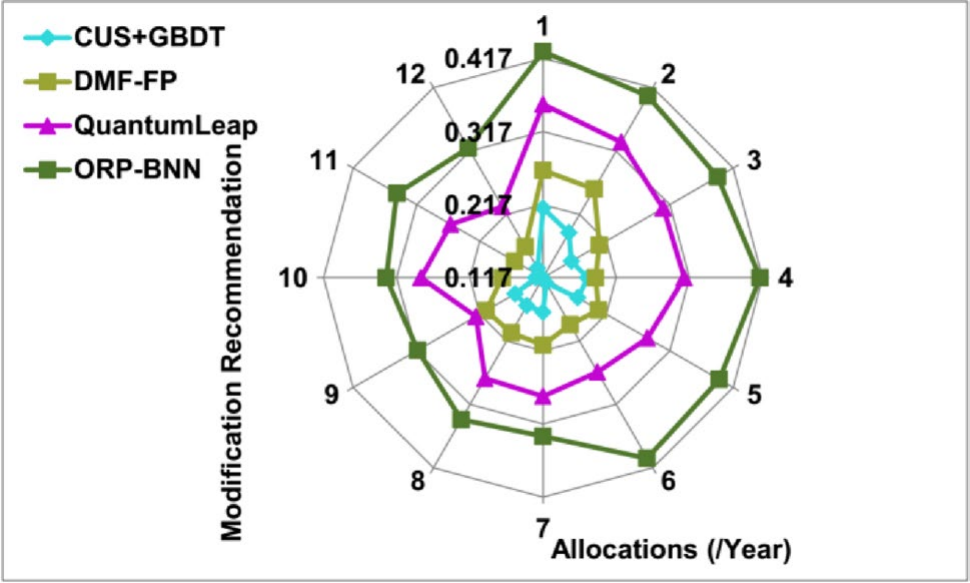
Modification recommendation analysis.

This snowfall model achieves high modification recommendation for fund balancing and risk prediction for maintaining corporate financial management; it depends on user needs, demands, and requirements for sustaining the corporate standards (Refer to Fig. 11). The model overhead and computing time is mitigated through assigning weight for all gradient loss identified in the computing model, whereas the financial structure rates and operations are modified. From the data input and corporate standard, the minimum riskless financial management is achieved through BNN. The fund allocation modification differs at the time of risk occurrence and overhead in organizations. The modification recommendation for improving the fund allocation and risk prediction is computed to address the flaws and overhead at the time of analyzing data. The risk occurrence identification is validated with pre-validation of existing and new financial imbalances computation for better fund allocation. Therefore, the overhead in this snowfall model is identified for increasing the financial management based on varying financial structures. Hence, the previous financial decision outcome depends on the high-risk weight of individual ${\rho }_{Org}$ and $Risk\, Prediction\left(N\right)$ balanced with each other.

### Computing time

**Figure 12:**
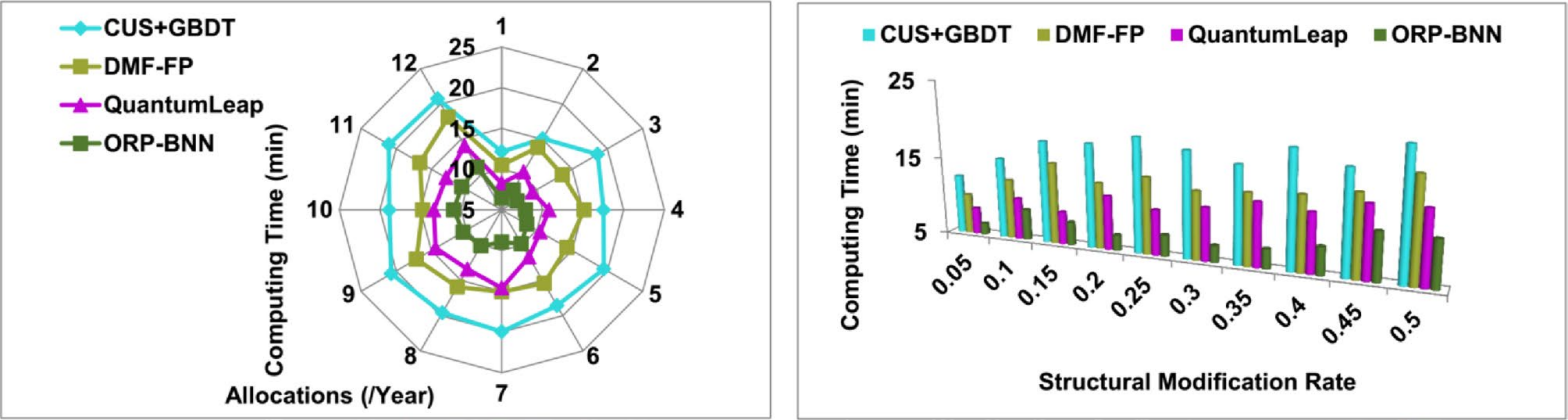
Computing time analysis.

In Fig. 12, the fund allocation and risk prediction are processed based on input data observed from the organization and analyzed using BNN and machine learning to validate the financial imbalance with the corporate standard. The risk prediction is performed by providing self-training through a snowfall model for modifying the financial structure. The minimum riskless financial management does not identify any risk at any organization through machine learning and computing model in different time intervals. The user needs and requirements-based changes are performed in fund allocation, and then modified financial structure and input data are used for finance optimization. The assigning significant weight achieves both the conditions of $N>t$ and ${\rho }_{{Org}_{n}}$ is computed constantly. The computing time and overhead are measured for risk prediction. The corporate financial management data analysis is performed to prevent flaws and overhead for the modification recommendation. The weight factor is determined using gradient loss functions associated with the modified new financial structure followed by the financial decision outcome. The available input data and financial structure is optimized relies on varying risk weight for which the proposed model satisfies less computing time.

### Model overhead

**Figure 13:**
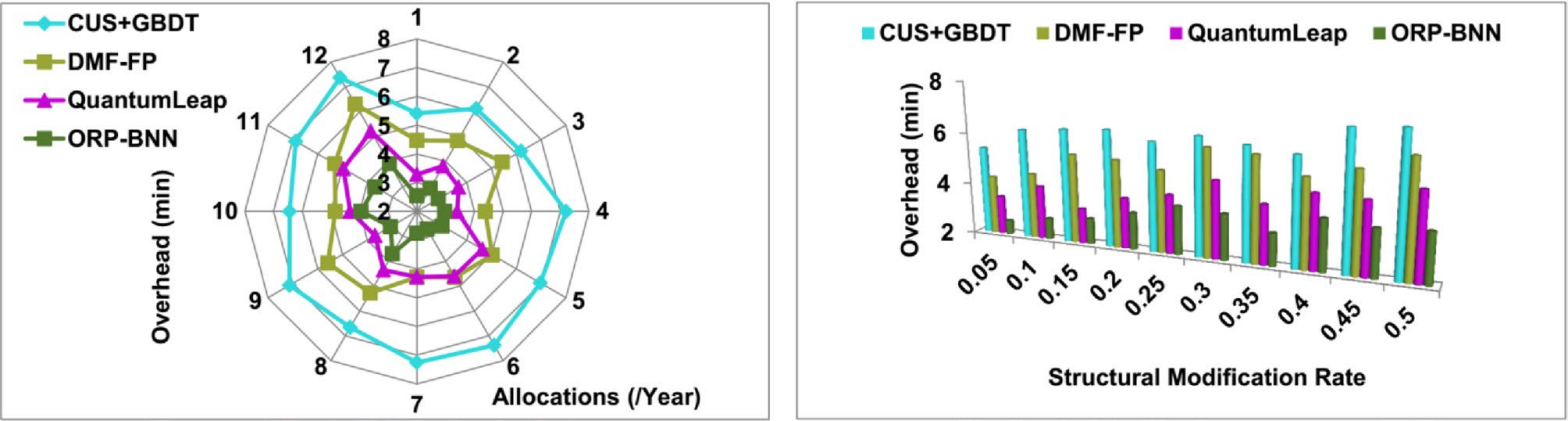
Model overhead analysis.

In Fig. 13, the finance optimization model depends on finance-related structures in single or multiple organizations and is analyzed for providing training using different structural modifications. The significance between fund allocation and restraining is deciding for financial imbalance verification. As per the input data analysis, the organizations jointly perform the self-training and external learning as it does not identify the investment and share risk at the time of fund allocation for managing better corporate standard. The user needs and demands are analyzed for improving the corporate financial management and training finance optimization for flawless and overhead-less fund allocation is processed. The risk thwarted financial planning relies on input data analysis and training in which both conditions are processed consecutively. The risk is addressed using weight factor and snowfall computing model instance if the data inputs observed from the different instance is used for training optimization. For the instance $\left({Inv}_{r}\right)$, $\left({Shr}_{r}\right)$ and $f\left(GrL\right)$ satisfies all the organization's financial structures, preventing risk factors.

### AUC-ROC analysis

ROC as a representation of risk management and assess the tradeoff between True Positive Rate (TPR) and False Positive Rate (TPR) with the effective financial risk management aims to balance the identification of real financial risks called losses while minimizing false alarms like non-existent risks using Eqs. (12) and (13).

12 $$TPR=No.\, of\, correctly\, predicted\, financial\, losses/Total\, no.\, of\, actual\, financial\, lossess$$

13 $$FPR=No.\, of\,  falsely\,  predicted\, financial\, losses\, \left(false\, alarms\right)/\begin{array}{c}Total\, no.\, of\,  actual\,  non-loss\,  scenarios\end{array}$$

**Figure 14:**
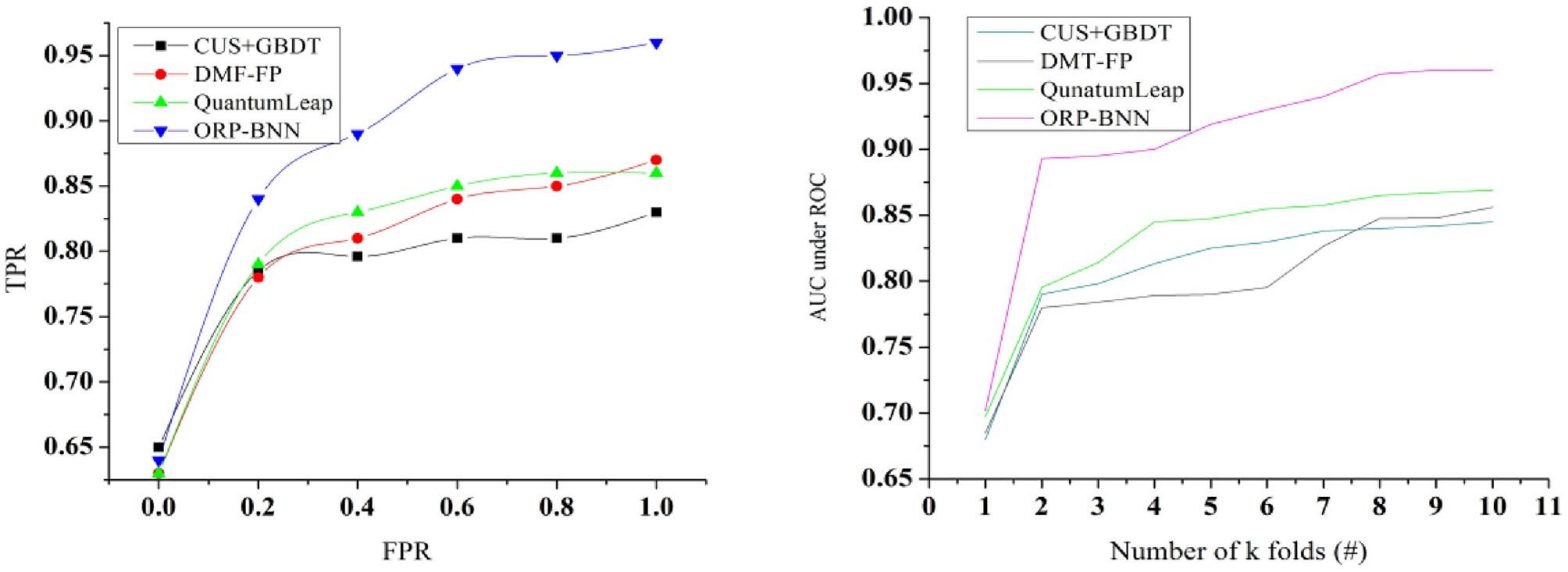
AUC-ROC analysis.

A financial manager’s decisions can be likened to adjusting the model’s threshold to optimize the tradeoff. AUC as an indicator of financial performance indicates the numerical value summarizing the performance of a classical model as an indicator of how well financial constraints manage their financial risks. As shown in Fig. 14 higher AUC in risk prediction suggests a better performance in identifying and mitigating risks, analogous to a financial entity’s ability to make decisions and optimize financial stability. The goal is to optimize the tradeoff between TPR and FPR to balance the identification of real financial risks (minimizing false negatives) while minimizing false alarms (minimizing false positives).

### Cross validation analysis

**Figure 15:**
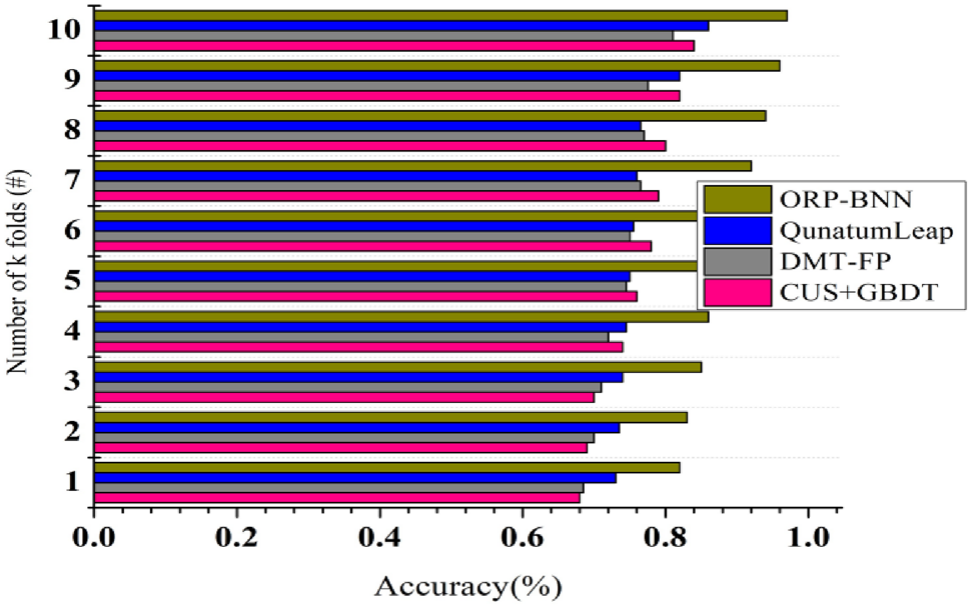
Cross validation vs model accuracy.

Cross-validation calculates the reliable estimate of the model’s performance and the range of performance score in terms of classification accuracy. It helps ensure that the model is robust and not overfitting to the financial data used during training period. Figure 15 shows that the k-fold cross validation data is divided in to k subsets or k folds where the model is trained k times, with each fold serving as the validation set once and the others as the training set. From these calculations it identifies the average performance of the model and how it varies across different subsets of the data. Cross-validation assesses how effectively the model generalizes to other data partitions like 30% random and 70% actual model, which aids in the detection of overfitting. The accuracy here represents the classification accuracy that represents the correct predictions regarding financial risks in terms of healthy and distress. The use of k-folds is to train the model for accurately classifying the financial risk. The performance metric for each iteration has been evaluated from the best on average across the folds with the training set and validate it on the validation set for each fold in k-fold cross validation.

The above discussion is summarized in Tables 1 and 2.

**Table 1:** Summary of allocations.

Metrics	CUS + GBDT	DMF-FP	QuantumLeap	ORP-BNN
Prediction rate	0.486	0.592	0.653	0.7489
Imbalance detection (%)	59.02	73.86	85.57	95.801
Modification recommendation	0.132	0.166	0.229	0.3218
Computing time (min)	20.75	18.15	14.09	11.045
Model overhead (min)	7.37	6.31	5.23	3.91

**Table 2:** Summary of modification rate.

Metrics	CUS + GBDT	DMF-FP	QuantumLeap	ORP-BNN
Prediction rate	0.436	0.575	0.652	0.7489
Imbalance detection (%)	53.12	72.37	83.51	95.815
Computing time (min)	21.01	17.86	14.16	10.88
Model overhead (min)	7.28	6.38	5.29	3.905

The proposed model improves the prediction rate, imbalance detection, and modification recommendation by 8.59%, 11.49%, and 14.6%, respectively. The proposed model reduces computing time and model overhead by 12.49% and 12.6%, respectively.

The proposed ORP-BNN improves prediction rate and imbalance detection by 9.73% and 13.07%, respectively. It reduces the computing time and model overhead by 12.81% and 12.72%, respectively.

## Conclusion

In this article, an optimal risk prediction model using a backpropagation neural network is designed to identify financial imbalance and organizational management risks. The proposed model is designed to analyze financial structures and recommend a precise modification based on financial imbalance. The imbalance is jointly identified using the backpropagation neural network and gradient loss function. The neural network is responsible for identifying different financial downfall probabilities. Considering the different factors such as investment, shares, and operations, fund allocation is planned using existing and previous successful models. The allocation and retaining decisions are performed using the BNN output that is further analyzed using a gradient loss function for precise computing. In this analysis, the computing model using gradient loss identifies unique downfall intervals wherein the corporate standard has to be maintained. This is required for identifying the first risks over consecutive intervals post the modification implication. Therefore, the finance model is well organized and planned for better outcomes across different investment options. This model is validated against snowfall preferences for different investment and market strategies. In the future work, a linear analysis model for successive quarter-based risk assessment and improvements are planned to be incorporated.

## Data Availability

All data generated or analysed during this study are included in this published article.
